# Digenic Inheritance of Hereditary Spherocytosis Type III and X-linked Agammaglobulinemia: Coexistence of Two Distinct Recessive Disorders in a Male Child

**DOI:** 10.7759/cureus.69887

**Published:** 2024-09-21

**Authors:** Khaled A Almutairy, Badriah G Alasmari, Syed Rayees

**Affiliations:** 1 Department of Allergy and Immunology, Prince Sultan Military Medical City, Riyadh, SAU; 2 Department of Pediatrics Hemato-Oncology, Armed Forces Hospital Southern Region, Khamis Mushait, SAU; 3 Department of Pediatrics, Armed Forces Hospital Southern Region, Khamis Mushait, SAU

**Keywords:** hereditary spherocytosis, immunology, pediatric hematology, recessive, x linked agammaglobinemia

## Abstract

Hereditary spherocytosis (HS) is a common inherited hemolytic disease caused by mutations in genes encoding proteins crucial to the red blood cell (RBC) membrane, leading to a change in RBC shape from biconcave to spherical. There are five distinct types of hereditary spherocytosis, with types III and V being autosomal recessive and types I, II, and IV autosomal dominant. X-linked agammaglobulinemia (XLA) is a common inborn error of immunity that impairs B cell maturation and differentiation. We report a case of a two-year-old Saudi boy with persistent anemia, recurrent chest infections, and a subgaleal abscess. A whole exome sequencing study revealed digenic inheritance of hereditary spherocytosis type III and XLA. Despite a variant of uncertain significance in the Bruton’s tyrosine kinase (BTK) gene, the patient's clinical and biochemical profile strongly indicated XLA. This case highlights how digenic inheritance can manifest as a complex phenotype, illustrating the challenges in diagnosing and managing patients with multigenic diseases.

## Introduction

Hereditary spherocytosis (HS) type III, an autosomal recessive disorder, affects the SPTA1 (alpha spectrin) gene on chromosome 1q23.1 [1]. This condition involves defects in the structural proteins of red blood cells (RBCs), reducing the surface area of the erythrocyte membrane and leading to spherical, hyperdense, and rigid RBCs. These abnormal cells have a shortened lifespan, resulting in anemia [2]. Severe recessive hereditary spherocytosis is primarily caused by biallelic mutations in the SPTA1 gene, with reduced SPTA1 mRNA expression in reticulocytes providing evidence of pathogenicity. The severity of the disease correlates with the level of α-spectrin protein in the RBC cytoskeleton [3,4]. X-linked agammaglobulinemia (XLA), or Bruton’s disease, is due to mutations in Bruton’s tyrosine kinase (BTK) gene on Xq21.33-q22. This disorder is characterized by frequent pyogenic infections, absence of B cells, and agammaglobulinemia [5]. Symptoms typically manifest after six months of age as maternal immunoglobulin levels decline, exposing the infant to recurrent bacterial infections, particularly from encapsulated bacteria such as Streptococcus pneumoniae, Pseudomonas aeruginosa, and Hemophilus influenzae. IVIG replacement therapy is essential for managing primary hypogammaglobulinemia, and the treatment adequacy is reflected in the spectrum of clinical symptoms [6,7].

## Case presentation

**Figure 1:**
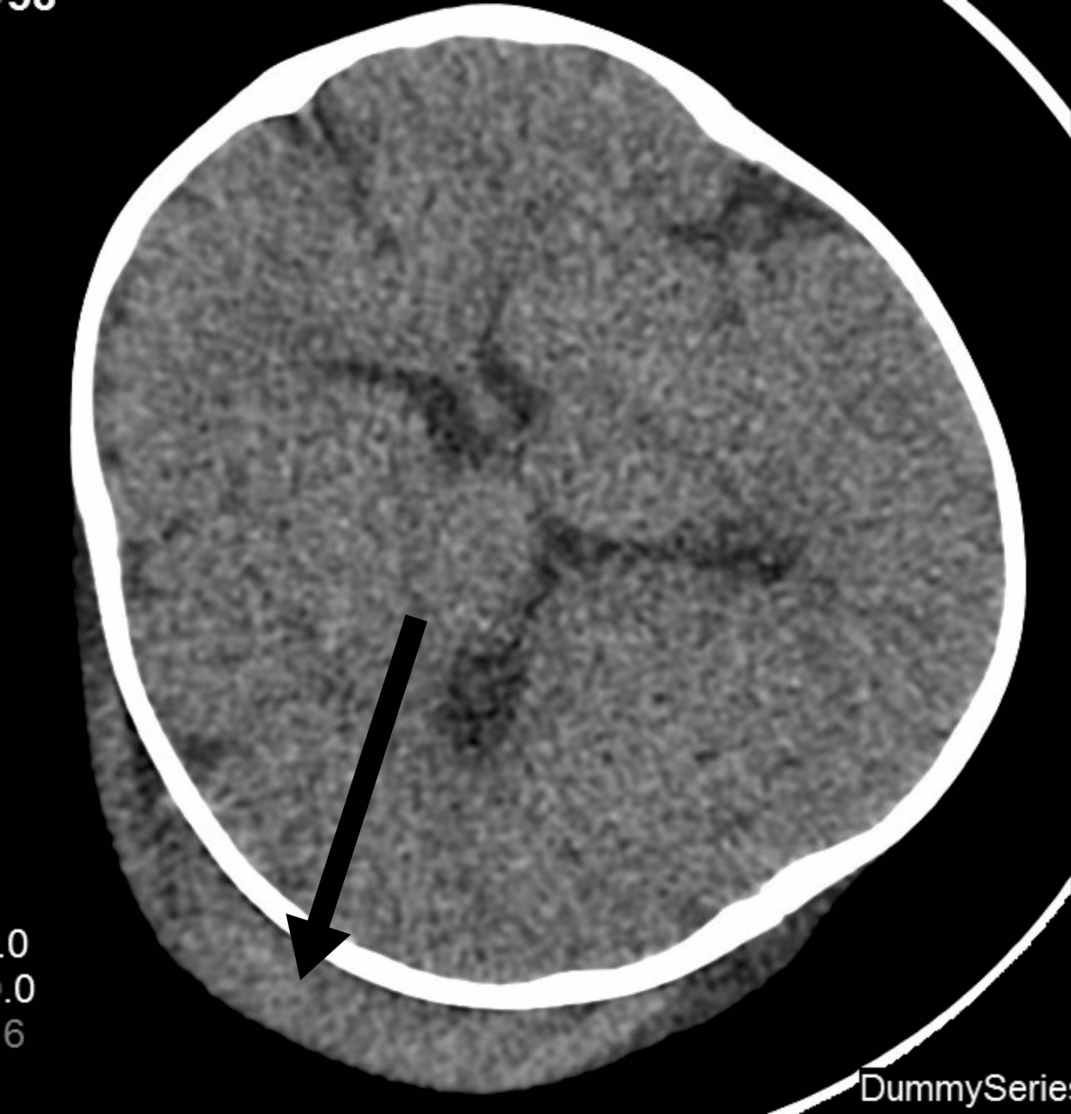
Axial CT scan of the brain revealed right parieto-occipital subgaleal hematoma (black arrow)

**Figure 2:**
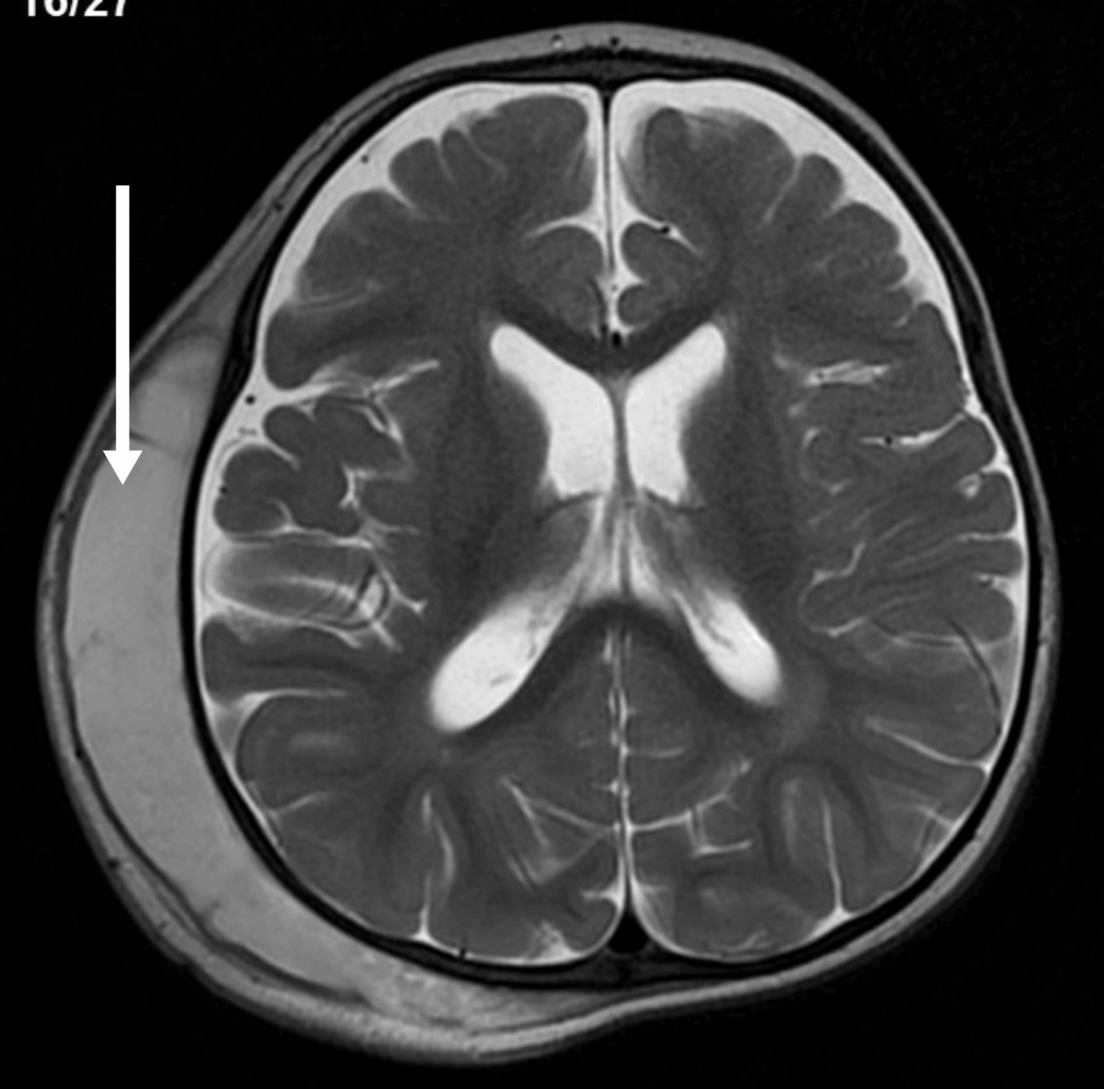
Post-contrast MRI of the brain: axial T2 WI MRI brain showed subgaleal fluid collections with bright signals and few septations

**Figure 3:**
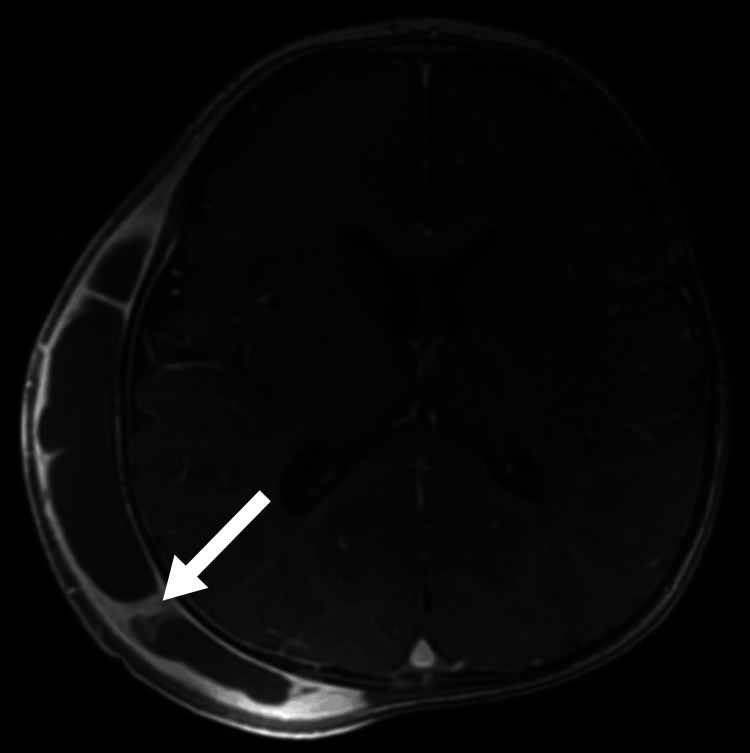
Post contrast axial T1 WI MRI of the brain revealed subgaleal fluid collection with markedly enhancing wall and internal septations

**Figure 4:**
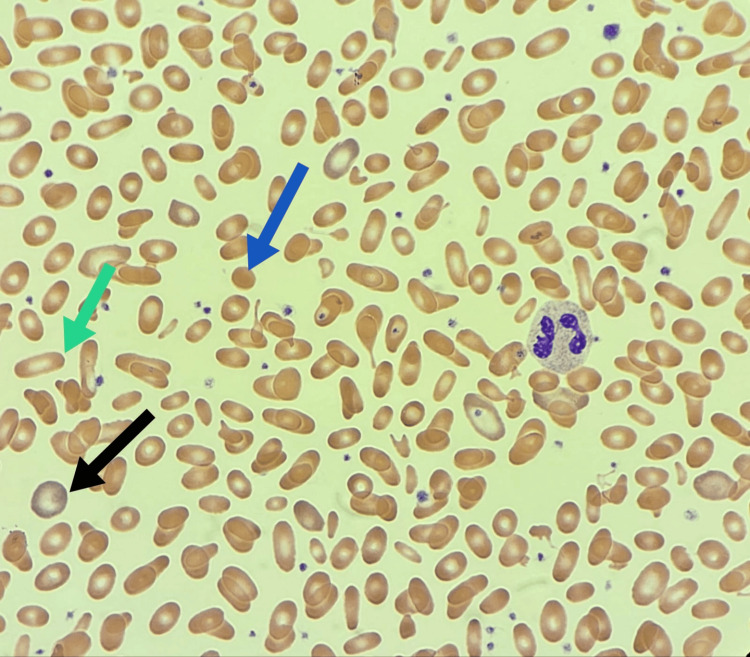
Peripheral blood smear 100× magnification Wright-Giemsa stain showing elliptocytes +3, hypochromic microcytic RBC +2, polychromasia +1, occasional micro-spherocytes, anisopoikilocytosis +3, adequate neutrophil, occasional atypical lymphocytes, no blasts. Mild thrombocytosis with normal-sized platelets. Green arrow: elliptocytes; Black arrow: polychromatic cells; Blue arrow: microspherocytes.

We describe a two-year-old Saudi boy born to consanguineous parents via cesarean section due to meconium-stained liquor and cord entanglement. Post-birth, he was treated for neonatal hyperbilirubinemia with phototherapy. Over the past year, he experienced five recurrent chest infections requiring hospitalization. At two years of age, he presented with persistent high-grade fever and swelling in the right parietal region following a head injury. A computed tomography (CT) scan (Figure 1) revealed a subgaleal hematoma and a small parietal bone fissure. MRI (Figures 2-3) confirmed an infected subgaleal hematoma, which was drained, revealing Proteus species in the abscess culture. Blood tests showed a normal coagulation profile and high-performance liquid chromatography (HPLC), elevated C-reactive protein levels and white blood cells, and microcytic hypochromic anemia. The hematology workup is outlined in Table 1. The peripheral smear revealed hypochromic microcytic elliptocytosis (Figure 4). Further investigations due to his anemia and recurrent infections revealed significantly low levels of immunoglobulins: IgM = <0.05 g/l (0.19-1.46 g/l), IgA = 0.0667 g/l (0.82-4.53 g/l), and IgG = 0.333 g/l (7.51-15.6 g/l). Lymphocyte subsets revealed a low B-cell count (Table 2), suggesting humoral immune deficiency.

**Table 1:** Hematology workup

Measured entity	Current value	Reference range
Hemoglobin	8 g/dl	10.9–15 g/dl
White blood cells	18.27 x 10^9^/L	5-15 x 10^9^/L
MCHC	29 g/dl	32–36 g/dl
Hematocrit	40.6%	31–41%
RBC	5.2 x 10^12^/L	4-5 x 10^12^/L
MCV	89.6 fl	70-86 fl
RDW	19%	11-14%
Platelets	500 x 10^9^/L	300-450 x 10^9^/L
Reticulocyte	7.2%	0.5–2.5%
Iron	11 μmol/L	12.5–32 μmol/L
Transferrin saturation	17%	<50

**Table 2:** Lymphocyte subsets

Hematology	Result	Reference range	Unit
Lymphocytes, total	2.80	2.70–11.90	tsd/μL
T lymphocytes (CD3+)	87	39–73	% lymph.
T lymphocytes (CD3+)	2430	1400–8000	/μL
T-helper cells (CD3+/CD4+)	1587	900–5500	/μL
T-helper cells (CD3+/CD4+)	57	25–50	% lymph.
T suppressor cells (CD3+/CD8+)	788	400–2300	/μL
T suppressor cells (CD3+/CD8+)	28	11–32	% lymph.
B-lymphocytes (CD19+)	14	600–3100	/μL
B-lymphocytes (CD19+)	<0.5	17–41	% lymph.
NK-cells (CD16+/CD56+)	307	100–1400	/μL
NK-cells (CD16+/CD56+)	11	3–16	% lymph.

Whole exome sequencing (Table 3) identified a pathogenic homozygous SPTA1 variant and a hemizygous BTK gene variant. The SPTA1 variant is a common disease-causing variant and has already been reported in multiple patients with hereditary spherocytosis. A functional test of this variant confirms that this mutation reduces the tetramer assembly and results in membrane destabilization. The BTK variant is not present in gnomAD or the NHLBI Exome Sequencing Project databases; hence, it is classified as a variant of uncertain significance. In silico programs (MutationTaster, Provean), a deleterious effect for this variant is suggested. The patient’s phenotype correlates with the deficiency of both BTK and SPTA1. Targeted molecular genetic testing and segregation analysis can be offered to the parents and other family members for further interpretation. In our case, the parents had refused segregation analysis.

The patient has been referred to a specialized immunology and bone marrow transplant unit and is currently on monthly IVIG therapy.

**Table 3:** Whole exome sequencing study MIM: Mendelian inheritance in man, AR: autosomal recessive, gnomAD: genome aggregation database, MAF: minor allele frequency

Gene (isoform)	Phenotype MIM number (mode of inheritance)	Variant	Zygosity	MAF gnomAD	Classification
SPTA1 (NM_003126.4)	270970 (AR)	c.7797T>C	Homozygous	0.0025	Pathogenic
	266140 (AR)				
BTK (NM_00061.2)	300755 (XL)	c.1905_1907dup	Hemizygous	0	Uncertain significance

## Discussion

Hereditary spherocytosis (HS) is a type of congenital hemolytic anemia that leads to mutations in the gene that encodes proteins that stabilize the erythrocyte membrane. The mode of inheritance is either autosomal dominant or autosomal recessive, with the former being the most common type. HS prevalence is higher in Northern Europe and North America, with 1 in 1,000-2000 affected [1,2]. Congenital hemolytic anemias encompass a heterogeneous group of rare hereditary diseases affecting erythrocytes that eventually lead to their reduced life span, causing anemia. Examples of congenital hemolytic diseases causing membrane defects include hereditary spherocytosis, hereditary elliptocytosis, and hereditary stomatocytosis [3]. These structural erythrocyte membrane defects lead to a decreased surface area of the red cell membrane that does not deform while passing through the small blood vessels, leading to its rupture. The erythrocyte membrane proteins include SPTA1 (α-spectrin), SPTB (β-spectrin), SLC4A1 (band 3), ANK1 (ankyrin), and EPB42 (protein 4.2). HS arises when any of these proteins fail to adhere the outer bilipid layer with the inner cytoskeleton, resulting in spherically shaped erythrocytes instead of the typical biconcave disc shape. This alteration diminishes the surface-to-volume relationship, leading to rigid erythrocytes that are fragile with decreased compliance to mechanical or osmotic stress [1]. Spherocytes are subsequently cleared from circulation during passage through the spleen, leading to hemolysis of spherocytes and anemia, which is reflected in decreased mean corpuscular volume (MCV), an increase in mean corpuscular hemoglobin concentration (MCHC), and increased osmotic fragility of red blood cells. A summary of the types of HS is listed in Table 4.

**Table 4:** Types of hereditary spherocytosis

Types of HS	Gene mutated	Protein affected	Inheritance	Gene location
HS 1	ANK1	Ankyrin	Dominant, de novo	8p11.21
HS 2	SPTB	Spectrin beta	Dominant	14q23.3
HS 3	SPTA	Spectrin alpha 1	Recessive	1q23.1
HS 4	SLC4A1	Band-3 Protein	Dominant	17q21.31
HS 5	EPB42	Protein 4.2	Recessive	15q15.2

The clinical severity of HS varies significantly, ranging from a mild form where individuals show no symptoms to a moderate form that presents with anemia, jaundice, and splenomegaly, and ultimately to a severe form that may involve a hemolytic crisis or in-utero hydrops fetalis.

Positioned at 1q22-23, the SPTA1 gene encodes α-spectrin, an essential component of the erythrocyte cytoskeleton that attaches to the plasma membrane surface via ankyrin and band 3. Recessive spectrin-deficient hereditary spherocytosis (HS) can be linked to mutations in the SPTA1 gene, whether homozygous or compound heterozygous. Heterozygous individuals can produce adequate α-spectrin to maintain the balance with β-spectrin and preserve the erythrocyte cytoskeleton. Most cases are attributed to missense and splicing mutations [4]. In our case, a homozygous variant was identified but without severe hematological symptoms.

XLA is a rare primary immunodeficiency disorder manifesting in the pediatric age and affecting males only due to its X-linked inheritance. This condition is characterized by the absence of B cells and a deficiency in immunoglobulins, specifically gamma globulins, leading to recurrent pyogenic infections. Otitis media, sinusitis, pneumonia, conjunctivitis, gastroenteritis, and skin infections like impetigo, cellulitis, abscess, and furuncles are common ailments that can affect these individuals [5]. Furthermore, these individuals may experience unusually small or absent lymph nodes, tonsils, and other lymphoid tissues. The absence of tonsil and neutropenia strongly suggests the presence of X-linked agammaglobulinemia, and these boys need to be evaluated at any time if they have recurrent infections. Recurrent upper respiratory tract infections are the most common presentation, followed by sinopulmonary, skin, gastrointestinal, and blood. The most common agent is Hemophilus influenzae type b (57.8%), whereas other agents are Streptococcus pneumoniae (15.7%), H. influenzae non-type b (15.5%), Pseudomonas aeruginosa (11.2%), and parasites (10%) [6]. Proteus is a Gram-negative bacillus and a facultative anaerobe, mostly causing urinary tract infections, nosocomial infections in immunocompromised individuals, and community-acquired infections. P. mirabilis causes CNS infections in neonates and immunocompromised children. Following the detection of a Proteus infection cultured from the subgaleal abscess in our patient, we conducted a detailed assessment of immunological markers, which indicated significantly diminished B cell levels. Low levels of immunoglobulin and B cells, ranging from mild to moderate, can be observed in some atypical XLA patients. To establish a definitive diagnosis of XLA when there is strong clinical suspicion, it is important to carry out a BTK gene analysis in addition to laboratory investigations.

Treatment for XLA patients typically involves the administration of replacement immunoglobulin and prophylactic antibiotics to mitigate the risk of infections. Immunoglobulin can be administered via two routes, either intravenously (IVIG) every two to four weeks or subcutaneously (SCIG) every 1-14 days [7].

Multigenic diseases, particularly those involving digenic inheritance, are increasingly acknowledged due to the widespread availability of next-generation sequencing, especially in populations with high rates of consanguineous marriages. Co-inheritance represents one model of digenic inheritance, where two separate diseases arise from mutations in two different genes, resulting in a unique phenotype like our patient's. Although the family segregation was not done, one would expect that both parents are heterozygous for the SPTA1 variant, and his mother is carrying the BTK mutation. This case illustrates the co-inheritance of two distinct genetic disorders, each characterized by a different Mendelian inheritance pattern, leading to an atypical phenotype. The clinical features align closely with the manifestations of hypogammaglobulinemia and reduced B cell counts associated with BTK deficiency, as well as the severe chronic anemia observed in SPTA1-related hereditary spherocytosis. Our report highlights the complexity of multigenic diseases and emphasizes the necessity of employing an impartial strategy for variant identification, particularly in individuals exhibiting unusual clinical features.

## Conclusions

It is imperative to conduct a comprehensive investigation for any patient experiencing persistent anemia rather than simply treating it as iron deficiency. Even in the absence of a family history of immunodeficiency, recurrent infections should raise concerns about potential immunodeficiency. With the progress of medical science, genetic studies should be employed in cases where the diagnosis is uncertain, enabling early intervention and preventing further damage. Patients with immunodeficiency should receive ongoing care at specialized centers, and their families should be referred to geneticists for counseling.
